# User Perceptions and Experiences of an Interactive Voice Response Mobile Phone Survey Pilot in Uganda: Qualitative Study

**DOI:** 10.2196/21671

**Published:** 2020-12-03

**Authors:** Raymond Tweheyo, Hannah Selig, Dustin G Gibson, George William Pariyo, Elizeus Rutebemberwa

**Affiliations:** 1 Department of Health Policy Planning and Management Makerere University School of Public Health Kampala Uganda; 2 Department of Public Health Lira University Lira Uganda; 3 Department of International Health Johns Hopkins Bloomberg School of Public Health Baltimore, MD United States

**Keywords:** interactive voice response, noncommunicable diseases, qualitative, Uganda

## Abstract

**Background:**

With the growing burden of noncommunicable diseases in low- and middle- income countries, the World Health Organization recommended a stepwise approach of surveillance for noncommunicable diseases. This is expensive to conduct on a frequent basis and using interactive voice response mobile phone surveys has been put forth as an alternative. However, there is limited evidence on how to design and deliver interactive voice response calls that are robust and acceptable to respondents.

**Objective:**

This study aimed to explore user perceptions and experiences of receiving and responding to an interactive voice response call in Uganda in order to adapt and refine the instrument prior to national deployment.

**Methods:**

A qualitative study design was used and comprised a locally translated audiorecorded interactive voice response survey delivered in 4 languages to 59 purposively selected participants' mobile phones in 5 survey rounds guided by data saturation. The interactive voice response survey had modules on sociodemographic characteristics, physical activity, fruit and vegetable consumption, diabetes, and hypertension. After the interactive voice response survey, study staff called participants back and used a semistructured interview to collect information on the participant’s perceptions of interactive voice response call audibility, instruction clarity, interview pace, language courtesy and appropriateness, the validity of questions, and the lottery incentive. Descriptive statistics were used for the interactive voice response survey, while a framework analysis was used to analyze qualitative data.

**Results:**

Key findings that favored interactive voice response survey participation or completion included preference for brief surveys of 10 minutes or shorter, preference for evening calls between 6 PM and 10 PM, preference for courteous language, and favorable perceptions of the lottery-type incentive. While key findings curtailing participation were suspicion about the caller’s identity, unclear voice, confusing skip patterns, difficulty with the phone interface such as for selecting inappropriate digits for both ordinary and smartphones, and poor network connectivity for remote and rural participants.

**Conclusions:**

Interactive voice response surveys should be as brief as possible and considerate of local preferences to increase completion rates. Caller credibility needs to be enhanced through either masking the caller or prior community mobilization. There is need to evaluate the preferred timing of interactive voice response calls, as the finding of evening call preference is inconclusive and might be contextual.

## Introduction

Low- and middle-income country populations suffer approximately 75% of all noncommunicable disease deaths annually (approximately 32 million deaths) [1,2]. Moreover, over 15 million of the noncommunicable disease deaths occurring in the low- and middle- income countries are premature (affecting people aged 30 to 69 years), accounting for about 85% of the global premature deaths from noncommunicable diseases [2].

The use of mobile phone surveys to collect data is expected to increase, leveraging the growing ownership of mobile phones in low- and middle- income countries, although the evidence of this utility is still limited [3,4]. Mobile phone surveys could complement existing noncommunicable disease risk factor surveys such as the World Health Organization (WHO) recommended stepwise approach for surveillance of noncommunicable diseases [5].

Interactive voice response (IVR) surveys are one type of mobile phone survey that could be used. IVR surveys use prerecorded audio files that ask participants to use the keypad on their mobile phone to answer questions. IVRs are mainly known for their use in customer service and public health work and have been used in the United States since the 1970s [6] but are also increasingly being used for continuity of patient health care beyond the hospital setting [7-9]. More recently in low- and middle- income countries, international development work has used IVRs, alongside other multimedia such as radio, with the former offering advantages for interactive reach to their audiences to stimulate behavior change [6].

IVR technology has been piloted and used mainly in health care settings in high-income countries dating back to the early 2000s [7,8], but IVR use is still limited in low- and middle- income countries [10,11]. Within high-income countries, IVR use is generally limited to exploring aspects of self-care [12,13], follow-up of patient care [7,14,15], and evaluating patient-provider interactions in clinical settings [9] but is rarely used for research or surveillance purposes [8,16,17]. Since mobile phone surveys are a relatively new methodology, particularly for low- and middle- income countries, evidence from community respondents on their perceptions on mobile phone surveys and possible reasons for taking the survey and nonresponse can contribute to better future mobile phone survey design and programming efforts.

A qualitative study [10] in Ghana that used focus groups to evaluate the experience of caregiver’s health care seeking for their sick child, based on receiving health information through an IVR, reported that all the 37 participants were naïve to IVR but held favorable perceptions about its use for symptom screening and providing guidance for care seeking. Negative perceptions included the fear for nonhuman interaction in using the IVR, a lack of familiarity with IVR, and the related cost [9,18]. Small-scale studies [19-22] have reported IVR use in low- and middle- income countries mainly for monitoring medication adherence, such as for tuberculosis and HIV. Within sub-Saharan Africa, sectors other than health, such as agriculture and social development have successfully used IVR for surveillance and community engagement [6,23].

It is unclear why some respondents complete surveillance questions using IVR and why some do not. We sought to explore user perceptions and experiences of receiving and responding to an interactive voice response mobile phone survey for noncommunicable disease risk factors, to inform the design and delivery of future surveys delivered using mobile phones.

## Methods

### Researcher Reflexivity

RT the first author was the interviewer for all interviews of this study, collected field notes, transcribed, and led the coding and data analysis. RT is a public health physician, a native of the country of this study. About a third of the participants were known to the researcher, while the rest were obtained through his networks.

### Study Design

A qualitative study design [24] was used to elicit the experiences of participants who had completed a structured interview on noncommunicable disease risk factors using an IVR survey delivered to respondents who owned or had access to a mobile phone [16]. This entailed call-backs to all the phone numbers of respondents to the initial automated IVR survey, irrespective of their response status. Those who answered and consented to being interviewed through follow-up calls delivered by a human caller were administered an in-depth interview over the phone to explore reasons for the initial response or nonresponse.

### Development and Adaptation of Survey Tools

The study deployed an adapted questionnaire based on an English-language version of questions selected by a joint team from the Johns Hopkins University, WHO, and the United States Centers for Disease Control and Prevention [16]. These questions had been derived from the WHO stepwise approach for surveillance of noncommunicable diseases survey [5], behavioral risk factor surveys [25,26], and the Tobacco Questions for Surveys [27]. The questions were adapted to the local Ugandan context and included local examples of fruits and vegetables, questions on smoking and tobacco use, alcohol consumption, physical activity, and history of checking for high blood pressure or blood glucose level. Although English is one of the official languages in Uganda, a significant portion of the population does not speak English, and in order to increase the reach of the survey, the adapted questionnaire was translated and back-translated into 3 of the 6 other major languages spoken in various regions of the country: Luganda, Runyakitara, and Luo. The 4 language versions of the questionnaire (including English) were digitally audiorecorded and loaded onto an IVR platform. The audiorecorded questionnaire had 69 items and was delivered to all participants via an IVR platform (Viamo).

The IVR platform was a software interface developed by a global social enterprise. The platform used connectivity through the local mobile network operators registered and active in Uganda and pregenerated random-digit dialing codes (such as 077XXXXXXX, or 070XXXXXXX) to dial across different mobile network operators, with a prerecorded voice IVR. The platform used a call-in number to randomly call across the network operators to access survey participants.

**Figure 1:**
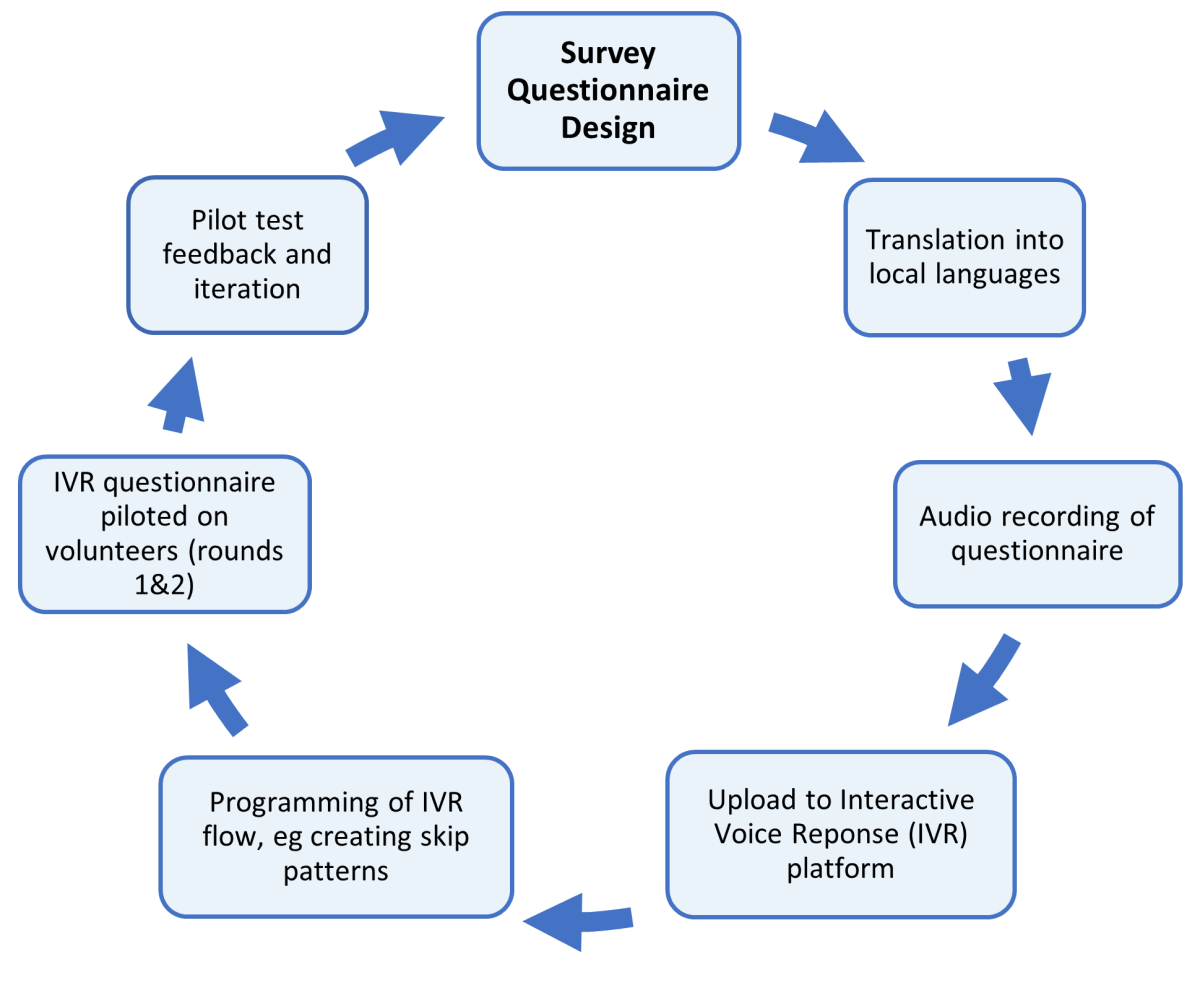
Process of IVR development and testing.

This platform delivery mechanism required pretesting to assess feasibility and aspects of acceptability. The survey also informed participants of a chance to win an airtime incentive (in a lottery) after completion of the IVR, as described elsewhere [28], specifically the possibility of winning the equivalent of US $0, $1.35, or $2.70. Figure 1 summarizes the process of conducting the pilot study prior to the main mobile phone survey.

### Study Population and Sample Size

The study population comprised adults who could understand or potentially speak any of the 4 languages in which the survey was deployed. Through contacts with communities in and around Kampala, Uganda. A purposive sample of 60 volunteers, was recruited to target 15 participants for each of the 4 languages of the questionnaire. The phone contact information of each of the 60 volunteers was uploaded onto the IVR platform, which then delivered an IVR call (up to 3 call attempts per testing round, if there was no answer). For example, surveys were programmed to call out at 4 PM, then for all unanswered calls, 2 hours and 4 hours later. Any incomplete IVR surveys, following the 3 attempts were not repeated. The IVR call was followed by a human caller to all the 60 volunteering participants, irrespective of their IVR response or completion status. The purpose of the human caller was to explore participants' feedback on their experiences with the IVR encounter and perceptions about the survey. Responses were recorded from 59 participants, representing a response rate of 98.3%.

### Data Collection

**Figure 2:**
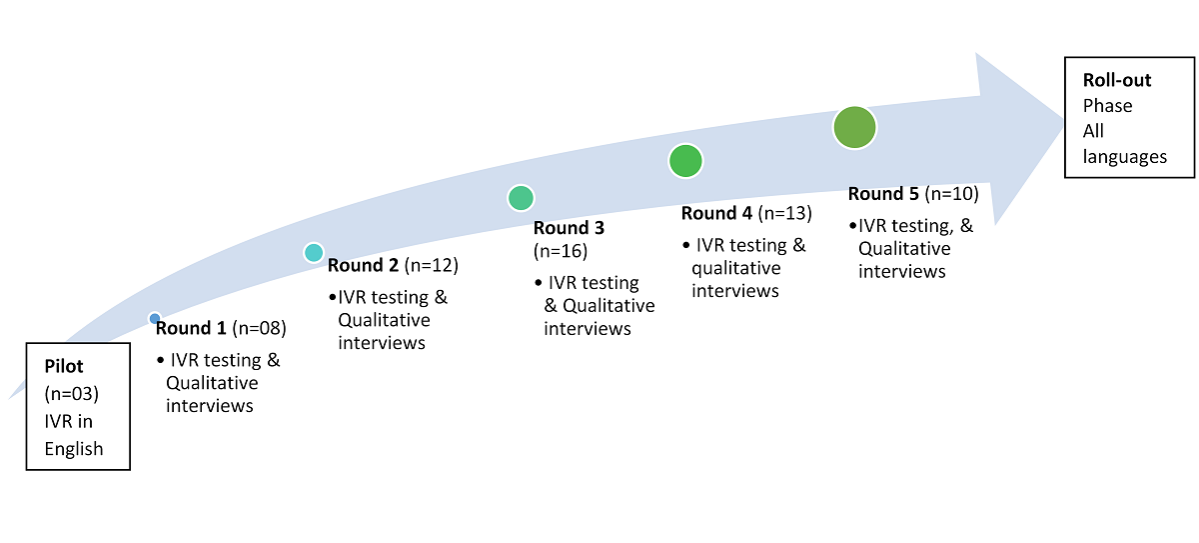
Data collection process.

The IVR survey and interview guide were pilot tested on 3 researchers in English prior to study deployment. Thereafter, as depicted in Figure 2, IVR survey testing and qualitative interviews were conducted iteratively in 5 rounds guided by data saturation. All rounds occurred in April and May 2018. There were a minimum of 2 native speakers taking the survey in each language in each of the first 3 rounds of the survey, designed to validate any differences in opinion for the same reported finding/ query from the survey. However, after 3 rounds of piloting, participants were selected purposively from within the network of the study coordinator and were not necessarily informed that they would receive a survey call, to mimic the real-life context in which prior survey booking may be impractical.

Following survey delivery, a research assistant called each IVR call recipient using the same language in which the IVR had been delivered and asked about their perception on whether or not the IVR was audible, if the subject in the questions was clear, if the pace was right, if the language was polite and courteous, and if the questions were understandable and appeared to be relevant based on the information provided at the beginning of the survey. Respondents were also asked about the difficulties they faced in receiving and navigating the survey, for example, if the instructions for responses such as pressing phone digits were comprehended, if they felt they were in control of the survey, and if they had any other feedback for the survey team. Survey testing rounds 1 to 5 were conducted in all languages, as depicted in Table 1.

**Table 1:** Characteristics of IVR survey participants (n=59).

Respondents		Interactive voice response round						Survey status		
		1	2	3	4	5	Completed survey		Failed^a^	Received (incomplete)^b^
n (%)		8 (13.6)	12 (20.3)	16 (27.1)	13 (22.0)	10 (16.9)	18 (30.5)		10 (17.0)	31 (52.5)
**Language**										
	English, n		●●	●●●	●		6		0	0
	Luganda, n	●●	●●●	●●●●	●●●●	●●●	3		3	10
	Runyakitara, n	●●●	●●●	●●●●	●●●●	●●●●	5		3	10
	Luo, n	●●●	●●●●	●●●●●	●●●●	●●●	4		4	11

^a^Cancelled or no answer.

^b^Wrong language selected: 7/31, 22.5% (English 0; Luganda 2; Runyakitara 1; Luo 4).

### Data and Theoretical Analysis Approach

A framework analysis, as first described by Ritchie and Spencer [29], was used to explore the themes [30] in the study related to audibility, question clarity, pacing of the study, language courtesy, and validity of questions. Framework analysis is advantageous in that it is purposive in nature (is not bounded to a specific epistemological position) guiding a researcher to identify themes that speak to specific objectives within a study, while exploring experiences within the narratives of participants [29-31]. In essence, both a priori coding from the objectives, and in vivo coding from emergent data are pursued in framework analysis [29,31]. Further to identifying themes within the study, we then explored for variability and the meaning of such divergent views using Janus-face theoretical constructs [32], thereby introducing postante codes to complement some apriori codes.

Janus-faced theory [32] (metaphorical perspective) on mobile phones was used to understand the interaction of participants with the mobile phone survey. The theory was chosen based on its simplicity for exploring distinct characteristics along the continuum from high- or low- interest regarding a naïve individual’s behavioral response while engaging with a technology [32]. We conceptualize the encounter of a naïve IVR user as likely to elicit a multiplicity of reactions, which may take the form of either acceptance or rejection of the IVR technology-interface. There were explanatory limitations for the use of potential alternative behavior change theories specifically, the Theory of Reasoned Action, The Theory of Planned Behavior, and the Social Exchange Theory [33-35]. Notably, the trio were limited in their assumption of and individual’s prior positive behavioral exposure, thus the choice of the Janus-faced theory [33-35].

The Janus-faces model proposed by Arnold [32] is derived from the metaphor of the Roman deity Janus who was cursed and blessed with 2 faces—each facing a different direction (backward and forward at the same time) [32,36]. The significance is that, while mobile phones and other technologies are designed and built to direct a specific purpose, in reality, a growing evidence base reports sociotechnical system of interaction findings that people’s reactions to technology, its use, and adoption can be ironic and paradoxical rather than unified and purposeful [36,37].

**Figure 3:**
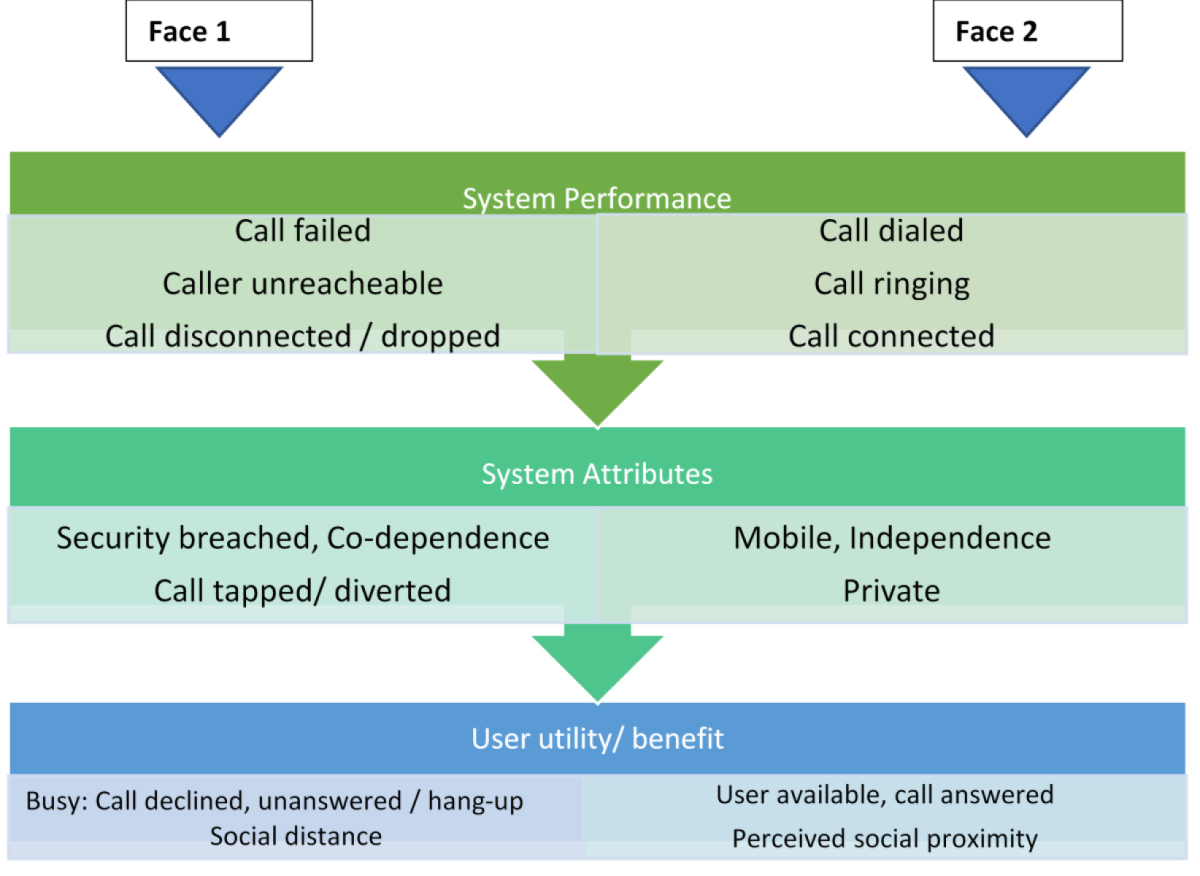
Janus-faced mobile phone utility.

An example of the theory's application to mobile phone utility and performance is presented in Figure 3. System performance criteria includes on the one-hand, issues such as call dialed, call ringing (reached), call connected, while on the other hand, it includes things such as—call failed, caller unreachable, call disconnected or dropped, which could be perceived by a user as either advantageous or not.

Ethics approvals were obtained from the Makerere University School of Public Health and the Uganda National Council for Science and Technology, while participant informed consent was embedded within the IVR. The process is published elsewhere [38].

## Results

### Survey Calls

Table 1 below shows the characteristics of respondents who took the IVR survey (including the language selected) and who later were interviewed qualitatively; 59 of the respondents provided feedback to the IVR and participated in the qualitative interviews. Nearly a half of the participants were female 44% (26/59), and the age ranged between 23 to 47 years (median age was 31 years and 35 years for female and male respondents, respectively).

### Qualitative Interview Findings

For the qualitative interviews, the majority of the respondents did not complete their surveys on the first attempt but rather on the second or third call attempt. However, in summary, and as depicted in Table 2, reasons that were stated for survey completion were related to the perceived credibility of the institution providing the survey (Makerere University); the fact that the survey was health-related; the clarity of questions, language, and instructions; being of short duration (10 minutes or less); and the possibility of winning an airtime incentive. On the other hand, the reasons reported for noncompletion were related to being busy, poor network connectivity, and suspicion because of the unknown identity of the caller.

The summary findings in Table 2 are synthesized according to 6 emergent themes related to the process of IVR survey delivery: timing of the survey, call quality, language-related issues, phone type used, survey duration, network connectivity, and perceptions on the incentives. Within each of these themes, we explore the concepts of overall experience with the survey, audibility, question and language clarity, courtesy and question validity. Furthermore, we contrast successful and unsuccessful encounters, from the provider standpoint of intended survey delivery.

**Table 2:** Summary of themes and issues related to survey completion or noncompletion.

Theme/issue	Observation/comment	Possible impact or implication
Timing of survey	Evening times between 6 PM and 10 PM were preferred. Wrong timing (day times), had lower completion rates.	Timing of IVR^a^ surveys should be evaluated for increasing reach and completion rate.
Call quality and credibility of the caller	Inaudibility and challenges with skip patterns compromised receiving calls. The credibility of the caller was paramount for motivating participation.	Platform programming should be tested and piloted for robustness prior to rolling out IVR surveys. Caller credibility should be ascertained in the IVR introduction.
Language-related issues	Language courtesy provided favorable IVR experiences, while wrong language selection affected validity of responses.	A double prompt for the selection of appropriate language is essential. Piloting should ensure courteous translations.
Phone type used	Both ordinary and smart phone users encountered similar challenges with IVR instructions, such as pressing the wrong digits.	User-technology interface is a barrier to the validity of IVR surveys, whose impact requires continuous evaluation.
Survey duration	Shorter realistic survey duration such as 10 minutes is preferred.	IVR surveys need not last longer than 10 minutes.
Mobile network connectivity	Rural respondents, and those located geographically distant from the Capital City had mobile connectivity challenges.	IVR surveys require additional strategies for reaching rural and remote populations, such oversampling.
Perceptions about the incentive	The lottery-type incentive for airtime was perceived favorably by participants	There is need to evaluate various IVR incentive thresholds to find which one increases survey completion.

^a^IVR: interactive voice response.

### Timing of the Survey

Several volunteer participants preferred their surveys in the evening (between 6 PM and 10 PM local time), because they were less busy then, off their day’s commitments.

It is very difficult for me to receive any call for a survey during the day, because I am not in charge of my schedule at work

P4 English

I saw the call, it actually came in twice, but I was in the field, and I merely ignored it. Maybe next time, if you call me in the evening, I may pick the call

P13 Runyakitara

In general, calls that were sent in the evening within the first 2 rounds of delivery had higher response rates than those sent out during the day (9/15 compared to 4/15 of the total 20 participants in the first 2 rounds including failed calls).

Once the call got received, some encounters (participant-phone interaction) were either successful—resulting in a completed call or were unsuccessful—resulting in early termination or nonacceptance. Details of call receipt are described in the subsequent phrases.

### Call Quality and Credibility of Caller

#### Successful Encounters

Successful call recipients were mostly those who received their calls in the evenings between 6 PM and 10 PM local time. Also, repeat calls had higher chances of acceptance compared to initial calls. Informing participants to expect the calls beforehand—although requested by some—did not seem to increase successful encounters. Also, from the final 2 rounds of the pilot, the majority of the recipients reported that a health-related survey from the Ministry of Health and Makerere University School of Public Health interested them and motivated their participation.

However, 2 participants who experienced an early termination of the survey, for unknown reasons, voiced dissatisfaction with the follow-up call because it commenced the interview afresh.

My survey stopped abruptly, but when I received the next call introducing the same survey, it just begun afresh, and this was really disappointing. Hmm, because it meant that I had to spend more time on your survey

P15 Luganda

#### Unsuccessful Encounters

Within the first round of pilot interviews, there were 4 main reported reasons for call failure, including missing calls due to wrong timing, inaudibility, challenges with the skip pattern, and also suspicion and skepticism as to the identity of the caller for some.

Several of the volunteer participants who missed their calls, requested to receive call backs later to take the survey. At least 17/39 participants made this request. A majority of the participants took their call during the second call attempt. Several participants explained that this was because they did not have the phone with them all the time. In fact, some participants shared their phones with other members of their household.

I am at home focusing on some other chores, after work, so I had forgotten that I had an important in-coming call, I am sorry

P7 Luganda

I saw the calls, they came in twice, but I did not know that I had to take the call. The caller number looked strange, it was not a usual call, so I thought it might be a conman from [location named], or another country

P26 Runyakitara

Eight call recipients (2 for each different language) reported inaudible calls during the first round of the pilot survey. They all struggled to listen in to make sense of what the survey was about, irrespective of the survey language. When we scrutinized these participants, some held ordinary mobile phones, while others used smart phones. However, all of them were in a rural setting, although based on routine phone calls, the network connectivity was fine. An alteration within the platform, improved audibility with the second and subsequent rounds of the pilot survey.

Two participants discussed the difficulties they encountered with skip patterns, which either altered the flow of the survey or curtailed their ability to complete the survey.

The tobacco screening question kept repeating itself, whether I pressed that I was a smoker, or not. It did not allow me to proceed to the next set of questions. Each time I punched in 1 or 3, it merely repeated the question until I was fed up and ended the survey

P9 English

The survey kept asking me what my age was, and as an example to enter 18 on the phone’s keyboard if my age was eighteen. However, each time I entered my age, it kept on asking me the same question

P16 English

Similarly, the initial calls for other languages other than Luo (English, Luganda, and Runyakitara) had challenges with skip patterns, such as automatically moving to the next question irrespective of the selection of a prompted answer option. These required altering from the programming side within the IVR platform, following which the challenge of skip patterns was resolved, for subsequent survey testing rounds. There were also varied experiences of IVR-mobile phone survey participation related to the language of delivery itself.

### Language-Related Issues

#### Successful Encounters

Within the first round of the pilot survey, 2 Luo speakers reported that their survey went very well. It was audible, it was clear, the timing was appropriate, the skip patterns worked very well, and the survey was easy to comprehend.

However, none of the participants who took the survey in any of the other 3 languages of the IVR described it as courteous. On the whole, after providing feedback to the recording studio and rerecording, the second round and subsequent rounds attained the required benchmark for language courtesy, question clarity, appropriate pace, audibility, and validity as gauged from the participant’s feedback.

#### Unsuccessful Encounters

At least 7 respondents reported taking the survey in the wrong language which they could not comprehend, although the introduction of the IVR provided for selecting an appropriate language option. The quote below exemplifies the challenge of selecting the wrong language.

I am a Swahili speaker, but I received the survey and took it in Luganda. I am not sure if my answer options were accurate or not

P33 Luganda

#### Mobile Network Connectivity

Limited clarity of survey questions was only consistently reported by participants who were in rural locations—either on the farm, in a University, or in homes that were more than 250 km distant from the Capital city. Their mobile phone survey was generally inaudible, and it self-terminated after a couple of attempts of replying. The survey team therefore interpreted this as due to poor mobile network connectivity.

#### Survey Duration

Three participants responding in English voiced strong opinions based on their experience with the IVR survey lasting about 10 minutes that the information about the survey’s duration in the introduction need to be altered from 20 to the realistic 10 minutes, to manage a participant’s expectations. When asked how long the survey took, the majority of the participants who had completed the survey responded:

...about ten minutes...

P7 English

Thus, resonating well with the experiences of those voicing the concern on survey duration.

#### Perceptions About Study Incentives

When asked about what they thought of the method of incentive, all the participants were pleased about the promised lottery-type incentive for receiving airtime. Some also reported that it encouraged them complete the survey, as they stood a chance of winning this incentive. Furthermore, it was reported by a few that this type of incentive in research was generally new to them, but it did not really matter.

## Discussion

### General

This pilot survey aimed to explore the perceptions of users of and nonresponses to the survey. The key findings that favored IVR survey participation or completion included preference for short surveys of 10 minutes or shorter, preference for evening calls between 6 PM and 10 PM, preference for courteous language, caller’s credibility, and favorable perceptions of the lottery-type incentive. While key findings curtailing participation or survey completion included if the voice was unclear, skip patterns were confusing, difficulties in interfacing with the phone to complete the survey, such as erroneous selection of digits for response options on both the ordinary and smart phones, suspicion about the caller’s identity, and poor network connectivity for remote and rural participants.

Most of the participants in this study preferred their IVR calls between 6 PM and 10 PM, suggesting a preference for calls outside normal working hours. Intuitively, late evening call preference is related to a period of limited interruption from the rest of the day’s competing demands. As reported from other studies [3,39,40], from a cultural perspective, it seems interruptive to receive a call while at work, especially if conducting formal work requiring team-effort, such as teaching in class, working in an operation theatre among others. In rural places where phone-charging is rationed to locations where there is power. It could be that phones are charged during part of the day making them inaccessible to a user, while in the evening the user catches up with missed phone calls. Similarly, if a phone is shared between a couple or household members, the individual that did not have it during the day might only have access in the evening when the phone holding partner or family member returns [3,39,40].

While for the majority, audible calls that were clear were received favorably, thus offering a promise to the acceptability of the mobile phone survey, the inaudible IVR calls, and those where skip patterns had errors compromised call completion. Considering that an IVR recipient requires to first listen to the voice call then to accurately interact with the phone to complete provided instructions. A high voice quality call that has simple and clear instructions is likely to maintain a respondent’s interest. Future IVR surveys require an extensive piloting phase to ensure the qualities of voice clarity, simplicity, nonambiguity, and respondent’s motivation or captivation for guaranteeing a successful IVR survey—as evidenced from other studies in sub-Saharan Africa decrying the IVR interaction [10,18-20].

Relatedly, a major finding in this study is that the quality of call reception (both the audibility and skip patterns) was related to programming challenges. In this IVR-mobile phone survey, the audios did not require rerecording, rather, an adjustment within the platform to increase their audibility. Likewise, the skip pattern errors were rectified within the platform, rather than with the audiorecording. This goes to confirm that in a software-based interface, programming, testing, and verifying appropriateness is important before roll-out of a software mobile health program, in this case the IVR-mobile phone survey platform. Contrary to the Janus-faced theory, which anticipates varied responses for each scenario [32,36,37], for the case of errors in the platform development, the resulting unintended errors elicited laborious encounters with the IVR survey for participants. Essentially, irrespective of the participant or their phone type, it appeared that errors in IVR delivery elicited annoyance and a poor experience with the IVR. Therefore, as with all communication strategies, piloting of IVR platforms (the communication channel) is important prior to mobile phone survey delivery for ensuring the expected quality of the IVR for recipients and the appropriate delivery of the intended message.

We found that caller credibility was crucially important as a motivator for survey participation. About a third of the participants reported that this survey from the Makerere University, with the Ministry of Health motivated their interest and participation. On the other hand, there was a sense of skepticism for some regarding responding to IVR (automated voice) calls because of fears of privacy—related to capturing individual’s identity, conmen—relating to potential fraud, and political interests that were unwelcome, as reported elsewhere [38]. This finding demands the prior sensitization of a community about planned research, including conducting community mobilization, an important prestep in routine house-to-house surveys such as the census. Regarding IVR-mobile phone surveys, considering the competing agents using the automated voice calls for information dissemination, or mobilization, an alternative of a prior SMS text message clarifying the intent of an IVR-mobile phone survey, the planned survey timelines, and clearly stating the authority sanctioning the survey will be useful for increasing survey participation. If resources are inadequate for prior community mobilization or SMS messaging, a viable alternative might be for caller masking, such as using a label “Health Survey from Organization X” instead of an identifiable caller phone number that is unfamiliar to recipients.

Perceiving the language as courteous motivated participation and survey completion. This finding appears related to the social connectivity with one’s local language—an important aspect of communication. Since IVR survey delivery mimics a human interaction, the quality of experience is important for motivating participation and completion, as reported in a Ghanaian study [10,18]. Therefore, language courtesy should be an important attribute considered in IVR survey development, piloting, and testing, prior to roll-out. Relatedly, although infrequent in this survey, taking the survey in an inappropriate language compromised the quality of survey responses. It might be that in a multilingual society such as Uganda, participants might not readily locate their preferred language in the IVR instructions. However, it is expected that this limitation will improve with increasing familiarity with IVRs, because respondents are not required to read and write but rather listen and act accordingly. Nonetheless, it is important for future IVR-mobile phone survey developers for multilingual settings to explore the extent of this problem—selecting the wrong language option. Also, a double-checking prompt would be useful to confirm that a given language is the appropriate choice.

Network connectivity was responsible in some instances for dropped calls. To explore the magnitude of this, a stratified analysis (for network operators) was conducted to assess the dropped calls. Among the 4 mobile phone providers, one had higher prevalence in the rural compared to others, and their call drop rate was considerably lower. Relatedly, the limited clarity of survey questions, alongside dropped calls was consistently reported by volunteers that were in rural locations—either on the farm, in a University, or in homes that are more than 250 km distant from the capital city. Their mobile phone survey was generally not too audible, and it self-terminated after a couple of attempts at talking back. We concluded that this was due to limited network connectivity. Mobile phone network coverage in Uganda is best in urban locations, likely due to economic motives of capturing high-density communities—thus depicting economies of scale. Therefore, developers and implementers of IVR surveys require strategies that capture rural populations, when representativeness is critical for answering survey objectives—such as considering oversampling of the rural remote populations.

Our finding for the preference for surveys lasting 10 minutes or shorter suggests existing competing work or leisure demands, thereby requiring shorter and precise mobile phone survey, as evidenced from the main Uganda IVR-mobile phone survey which lasted an average of 13 minutes, yet with a low completion rate of 35.2% [28]. While routine face-to-face surveys such as the Demographic and Health Survey conducted every 5 years in low- and middle- income countries may last an average of 1 hour. The absence of physical human interaction in the IVR encounter tends to remove the normative desire of avoiding disappointing the interviewer, lest the participant be judged as rude—frontstage, back-stage acting. IVR participants are in control of their survey’s continuation or termination, which might shield their fears for potential retribution. Also, the lack of human interaction negates the opportunity to negotiate the timing and duration of the IVR. Therefore, commencing the IVR might somewhat rely on the curiosity of a respondent wanting to discover what the survey is about. However, considering that the average survey duration was about 10 minutes in this pilot, and there were no complaints that it took very long, this finding strongly implies that IVRs require brevity to maintain the interest of participants.

### Conclusions and Recommendations

Our findings show the willingness of participants to take an IVR survey. Key attributes of an IVR survey with promise for high uptake and completion within a multilingual context include: a preference for evening calls, of high voice quality and clear instructions, lasting 10 minutes or shorter, from a credible caller, and in a courteous language.

Findings emphasize the need for extensive platform development in the testing period to ensure stability, prior to roll-out of an IVR survey. There is need to further evaluate these attributes to increase IVR acceptability and completion rates in such settings.

It appears from our findings that both ordinary and smartphone users encounter interactive challenges with an IVR call, thus emphasizing a need for education of the community on use of IVRs. Suspicion as to the credibility of the survey authority suggests a need for caller masking. There is need for further research to explore reasons for low completion rates of IVRs compared to face-to-face surveys and whether language selection and education status affect the quality of surveys.

### Study Limitations

While this study used a qualitative methodology, user perceptions on IVR and nonresponse were collected through a phone interview and were not validated physically, which within the context of the study could have introduced some sociodesirability bias; however, phone interviews are a widely accepted method in qualitative research [24].

The pilot was limited to 3 nationally representative languages in addition to English, although 4 languages would have been more representative. Being an explorative study, the nuances from the findings might apply to the rest of the country, given that the cultural context is similar.

Additionally, at least a third of the participants were known to the first author who did the recruitment. This could have positively affected participation in the IVR and the qualitative interviews. However, both procedures followed standard research ethical practice after obtaining informed consent.

## Abbreviations

HIV: human immunodeficiency virus
IVR: interactive voice response
SMS: short message service
WHO: World Health Organization
